# A Mechanistic Account of Wide Computationalism

**DOI:** 10.1007/s13164-016-0322-3

**Published:** 2016-10-24

**Authors:** Luke Kersten

**Affiliations:** University of Edinburgh, Edinburgh, UK

## Abstract

The assumption that psychological states and processes are computational in character pervades much of cognitive science, what many call the computational theory of mind. In addition to occupying a central place in cognitive science, the computational theory of mind has also had a second life supporting “individualism”, the view that psychological states should be taxonomized so as to supervene only on the intrinsic, physical properties of individuals. One response to individualism has been to raise the prospect of “wide computational systems”, in which some computational units are instantiated outside the individual. “Wide computationalism” attempts to sever the link between individualism and computational psychology by enlarging the concept of computation. However, in spite of its potential interest to cognitive science, wide computationalism has received little attention in philosophy of mind and cognitive science. This paper aims to revisit the prospect of wide computationalism. It is argued that by appropriating a mechanistic conception of computation wide computationalism can overcome several issues that plague initial formulations. The aim is to show that cognitive science has overlooked an important and viable option in computational psychology. The paper marshals empirical support and responds to possible objections.

## Introduction

Jerry Fodor once claimed that: “quite independent of one’s assumptions about the details of psychological theories of cognition, their general structure presupposes underlying computational processes” (1975, p.28). Fast forward 30 years and views have changed little. Paul Thagard, for example, writes: “[t]he central hypothesis of cognitive science is that thinking can best be understood in terms of representational structures in the mind and computational procedures that operate on those structures” (2010, p.6). Suffice it to say, the assumption that psychological states and processes are computational in character pervades much of cognitive science, what many call the “computational theory of mind”.

Yet in addition to occupying a central place in cognitive science, the computational theory of mind has also had a second life supporting “individualism”, the view that psychological states should be taxonomized so as to supervene only on the intrinsic, physical properties of individuals (Fodor 1980, 1987; Stich 1983; Egan 1992). One route to individualistic psychology is what Robert Wilson calls the “computational argument for individualism.” Wilson (1994, p.353) formulates the argument as follows:

1. Cognitive psychology taxonomically individuates mental states and processes only qua computational states and processes.
2. The computational states and processes that an individual instantiates supervene on the intrinsic, physical states of that individual.
3. Therefore, Cognitive psychology individuates only states and processes that supervene on the intrinsic, physical states of the individual who instantiates those states and processes.

One response to the computational argument has been to challenge premise (2). This is the route adopted by Wilson (1994, 1995). Wilson argues that since not all computational processes are instantiated in the head, not all psychology is individualistic. Wilson raises the prospect of “wide computational systems”, in which some computational units are instantiated outside the individual. By enlarging the concept of computation, Wilson attempts to sever the link between individualism and computational psychology.

The idea of “wide computationalism” is more than a little interesting. Not only does it represent a substantial departure from orthodox thinking in cognitive science (Marr 1982; Pylyshyn 1984), but it also offers distinct grounds for thinking about extended cognition (Clark and Chalmers 1998; Rowlands 1999; Wilson 2004; Wilson and Clark 2009). However, for one reason or another, wide computationalism has received little attention in philosophy of mind and cognitive science. Though there has been some scattered discussion, no sustained analysis has been offered. This paper aims to revisit the prospect of wide computationalism.

The problem is that several issues plague initial formulations of wide computationalism. For this reason, focus here is given to buttressing the view via recent discussions of “mechanistic” computation (Piccinini 2007, 2015; Milkowski 2015). The argument is that by appropriating a mechanistic conception of computation the problems that emerge for earlier formulations of wide computationalism can be avoided (sections 3 and 4). The goal is to show that cognitive science has overlooked an important and viable option in computational psychology. On route to this conclusion, the paper marshals empirical support for “wide mechanistic computation” and responds to possible objections (sections 5 and 6).

A quick clarification is in order before discussion gets going. Wide computationalism, as Wilson presents the view and how it is developed here, is not meant as a global thesis. It does not imply that all computational systems are instantiated, at least in part, outside the body. Rather, the view is better understood as a supplement to individualistic psychology. It is an extension of the logic of computational analysis in cognitive science, rather than a replacement.

## Wide Computationalism

In the abstract, wide computationalism gains a foothold via the location neutrality of computational individuation. Because the method of computational analysis is noncommittal about the kinds of physical states that might be computationally characterized, it is at least possible that some of the relevant computational states might reside outside the individual. Wilson, for instance, writes: “There is nothing in the method of computational individuation itself…which implies that the class of physical features mapped by a realization function cannot include members that are part of the environment of the individual” (1994, p.355). Since formal systems are indifferent to physical medium and computation is a formal system, it is at least possible that some computational states and processes reside outside the individual.

The location neutrality of computational analysis also carries with it implications for psychological theorizing. This is because if psychological states and processes are computational in character and computational analysis is location neutral, then some psychological states or processes may extend beyond the boundary of the individual. As Hutchins points out in his discussion of Marr (1982): “Marr intended his [computational] framework to be applied to cognitive processes that take place inside an individual, but there is no reason, in principle, to confine it to such a narrow conception of cognition” (1995, p.50). The allegiance of psychology to computational theory carries with it the potential for extended cognition (see, e.g., Hutchins 1995; Wilson 2004; Kersten 2015; Kersten and Wilson 2016). Whether or not computational cognitive systems are instantiated exclusively within the boundary of the individual is an a posteriori question.

Wilson furthers develops wide computationalism by outlining a method for identifying wide systems. He writes: “The account of actual implementation is a generalization of that in the case of narrow computational systems: a wide computational system implements the ‘program’ physically stored in the environment with which it causally interacts” (1994, p.360). Similar to identifying “narrow” (in-the-head) computational systems, wide computational systems are present if there is a computational description that tracks causal transitions running from an organism’s environment to its internal physical states.

Interestingly, in identifying wide systems in this way Wilson aligns wide computationalism with “causal mapping accounts” of computation (Chrisley 1995; Chalmers 1994, 1996; Scheutz 1999, 2001). Accounts of this stripe claim that for a physical system to perform a computation there must be a mapping from a subset of states ascribed to a physical system by a physical description to states defined by a computational description. For any computational state transition of the form S_1_ → S_2_, if a system is in a physical state that maps onto S_1_, then the physical state that maps to S_1_ must cause the system to go into a further physical state that maps onto S_2_ (see Piccinini 2015, ch.2). Causal mapping accounts articulate the conditions for ascription of computational implementation in terms of isomorphic mappings between computational descriptions and physical descriptions via transitions between physical states.^1^

Commitment to the causal mapping account is more fully revealed in Wilson’s comment that: “[f]or a physical device to be capable of implementing a given program [computation] is for it to have its physical states configured in such a way that transitions between those states are isomorphic to transitions between states that the program specifies” (1994, p.360). The identification of actual computations, whether narrow or wide, requires mapping computational states to physical states in virtue of tracking causal relations between physical states.

Proponents of wide computationalism not only think that the notion is coherent, but that some organisms actually implement wide computational systems. Wilson, for instance, offers two examples of what he takes to be wide computational systems.

The first is Sekuler and Blake’s (1990) multiple spatial channels theory of vision and form perception. The crucial feature of this account is that specific sets of neurons are sensitive to specific sets of stimuli. These stimuli are decomposable into sinusoidal gratings with four parameters: spatial frequency, contrast, orientation, and spatial phase. Any natural scene can be decomposed into these formal primitives. For Sekuler and Blake, perception is the result of organisms processing environmental inputs through spatial channels and turning them into complex internal representations.

What is crucial about this example for Wilson is that the account acknowledges the computational role states beyond the individual play within perceptual processes. The computational analysis involves, first, identifying and describing the formal primitives instantiated in the physical environment and, second, describing how such inputs function to produce internal representations. Instead of viewing computation as beginning at the retina and ending at the visual cortex, Wilson maintains that computational analysis begins further downstream in the environment of the perceiving organism. Framing things in terms of the causal mapping account, the claim is that there is a computational description amenable to the physical states internal and external to the organism that help to explain form perception.

It is also worth noting that although there is sometimes a tendency in discussions of extended cognition to devalue or even dismiss the need for internal representations, there is nothing strictly antithetical about the two notions (see, e.g., Wilson 2004; Clark 2008). Wide computational systems of the kind relevant to extended cognition can trade in internal information-bearing vehicles just as easily as they can external ones. What is important is the role internal or external information-bearing vehicles play in the larger computational analysis. The appeal to internal representations in Wilson’s examples is simply the logical extension of pushing computational analysis further out into the environment.^2^

The second example Wilson offers is Gallistel’s (1989a, 1989b) account of animal spatial navigation. Wilson points out that according to Gallistel’s theory, animals construct complex representations of their environments in order to guide behaviour by instantiating modules sensitive to formal geometric structures of the environment. One example of this process is dead reckoning in ants and bees. These organisms take as their inputs three features: the animal’s solar heading, forward speed and a representation of the solar azimuth. What they produce is a representation of position relative to some landmark. There is a physical process characterizable as a computational process that begins in the environment and ends in the organisms, specifically as a representation of relative position. Framed again in terms of the causal mapping account, there is a causal transition between physical states isomorphically related to a computational description of spatial orientation. There is a subset of physical states that track transitions between computational state descriptions.

A third example of wide computation comes from Edward Hutchins’ (1995) work on navigation. Hutchins contends that when members of a navigation team carry out coordinating actions in service of a larger task – for example, navigating a ship at sea – they form a larger computational system that transcends the individual team members. Navigation – that is, the task of figuring out where something is relative to other positions – is achieved not only through the local actions of individual team members, such as the Navigator or Fathometer Operator, but also through the coordinated activity of the team members as a whole. Hutchins, for instance, writes: “In their communication and in their joint actions, the members of the navigation team superimpose themselves on the network of material computational tools of the trade” (1995, p.219). The navigational team can be thought of as a wide computational system because the functional whole extends beyond the local actions of individual team members. The social organization becomes the computational architecture on which the larger functional task is carried out.

Putting aside minor differences among the examples for the moment, the central message is that wide computational systems are not only theoretically possible, but that they are physically implemented in a number of cases. 3 For authors such as Wilson and Hutchins, the fact that research in human and animal psychology provide putative examples of concrete computational systems beyond the boundary of the individual is further vindication of the idea that at least some portion of computational psychology is not individualistic.

## Concrete Computation

One of the main problems for computational theories of mind is the problem of “computational implementation” (Chalmers 1994, 1996, 2011; Sprevak 2012). The issue is one of how to specify the conditions under which computations can be said to take place in physical systems. If psychological states and processes are computational in structure, then any successful account of computation has to explain how those computational states and processes are instantiated in physical systems. Without a successful account of computational implementation, the chances of a robust computational cognitive science diminish.

Several proposals have been offered, perhaps most famously Putnam’s (1967) mapping account. Of interest here is the solution offered by wide computationalism. Recall that wide computationalism subscribed to the causal mapping account of computation. For the causal mapping account, concrete computations occur just in those cases where there is a mapping of computational to physical descriptions tracking causal transitions between physical states. By specifying the isomorphic relations between physical states and computational states via causal transitions, the causal mapping account provides a resolution to the problem of implementation, and thus so too does wide computationalism.

However, a solution to the problem computational implementation is only one desideratum on an account of computation. This is because not only should an account of computation explain how physical systems compute it should also do justice to the sciences of computation. It should strive to do right by the practices of computer scientists, engineers, and cognitive scientists. Piccinini (2015, ch.1) highlights six such desiderata:

1. *Objectivity*. The account should make whether a system performs a particular computation a matter of fact. It should establish some form of objectivity on questions of computational implementation.
2. *Explanation*. The account should explain the behaviour of computing systems in terms of the procedures being executed. It should say how appeals to program execution, and more generally to computation, explain the behavior of computing systems.
3. *The right things compute*. The account should include the paradigmatic examples of computing mechanisms, e.g., finite state automata, Universal Turing machines, etc.
4. *The wrong things don’t compute*. The account should not entail paradigmatic examples of non-computing mechanisms, e.g., galaxies, digestive systems, etc.
5. *Miscomputation*. The account should explain how computations can go wrong.
6. *Taxonomy*. The account should provide a taxonomy that is able to distinguish between different kinds of computing machines, e.g., finite state automata, Universal Turing machines, calculators, etc.

The question is whether wide computationalism, as a computational theory of mind, satisfies these six desiderata. If it does not, then it may not be a viable account of computational cognition.

First, does wide computationalism provide some form of objectivity about computation? By endorsing the causal mapping account, it would appear so. Wide computationalism restricts the class of physical systems that can be said to implement computations by admitting only those systems that map computational descriptions to physical state transitions.^4^ Deciding whether or not a system computes is in some sense a matter of fact according to wide computationalism.

Second, does wide computationalism account for how program execution explains computing behaviour? Here the account stumbles. To qualify as a computational explanation, wide computationalism must explain how a program generates a system’s behaviour via a rule or program.^5^ The problem is that although wide computationalism describes where a program is represented by causal transitions (for example, the *ephemeris function* in the case of animal spatial navigation), it does not show how physical systems deploy or execute computational programs in the production of behaviour. Although the causal mapping account provides a computational model or description, it does not offer a computational explanation.

With respect to the third desideratum, wide computationalism performs satisfactorily. This is because it entails that there is some subset of physical states in most computing devices – for example, digital computers and calculators – that can be mapped to computational descriptions. Under a wide computational rubric, it is possible for each paradigmatic computing device to map from some of its physical states to the relevant computational states.

The fourth desideratum is not so easily met. On Wilson’s formulation, physical states of paradigmatic non-computational processes, such as the weather or respiration, can also be mapped to computational descriptions. The problem is that non-paradigmatic cases also trade in the right kind of causal transitions such that they can be mapped to computational state descriptions. Although it manages to account for cognitive systems and computers, wide computationalism also problematically entails that physical systems that should otherwise not count as computing systems nonetheless qualify. In short, the causal mapping account underwriting wide computationalism is too liberal.

Fifth, does wide computationalism account for miscomputation? Here, again, the account stumbles. Miscomputation requires that a computational system deliver the wrong output. In the case of a wide computational system, this entails that, for example, with respect an animal’s spatial navigation system in Wilson’s second example, a wide computational process could be mapped to the wrong output. But this does not appear possible given the causal-mapping account. In each case of wide computation, the computational description will map to the correct physical states (e.g., the animal’s solar heading, forward speed and a representation of the solar azimuth). The account cannot but deliver the correct relative position. Part of the problem is that causal mapping accounts can be generated regardless of whether a computation produces the correct result.

Finally, does wide computationalism provide a taxonomy of computing devices? In line with its handling of desideratum (1), wide computationalism does seem to be able, at least in principle, to furnish a categorization of computing mechanisms on the basis of casual powers. This is because only some systems will support physical transitions that can be mapped to a computational description. Wide computationalism provides enough matter of fact about which physical systems support computations to distinguish between the powers of different computing systems.

In sum, as an account of concrete computation, wide computationalism is strong with respect to desiderata (1), (3), and (6), but weak with respect to (2), (4), and (5). Not a bad performance, but things could be better. Part of the reason for this less than ideal showing is the view’s allegiance to the causal mapping account. The weaknesses of the casual mapping account carry through to wide computationalism. More positively, the prognosis is that if the underlying account of computation can be updated, then this may supply wide computationalism with the resources to address the outstanding desiderata, ultimately saving it from the dustbin of promising but unworkable ideas.

## Mechanistic Computation

Gualtiero Piccinini (2007, 2015) has recently laid out an account of physical computation that he dubs “the mechanist account” (see also Milkowski 2015). In what follows, I argue that the mechanistic account can be used to update the conceptual foundations of wide computationalism. This update allows the view to meet the three desiderata that caused trouble for earlier formulations.

Piccinini outlines three conditions for concrete computation. The first is that physical computing systems must be kinds of functional mechanisms. The system has to possess properties that organize in such a way so as to produce or support some behaviour – the reverse of which is that if a system fails to perform its function it must be the result of a breakdown in the organization of the system’s component parts.

The second condition is that one of the capacities of a mechanism must be the ability to compute at least one mathematical function. The system must be able to map from an input I (and possibly internal states S) to an output O. The system’s behaviour must satisfy at least one abstract description mapping inputs to outputs – this also suffices to show that the system is following a rule.

The final condition is that a physical computing system must compute its function via the manipulation of medium-independent vehicles. This means that informational vehicles – whether they are numbers, symbols, or retinal images – must be transformed over the course of a computation in virtue of a system’s sensitivity to some part of the vehicle’s structure. So, for example, in the case of numbers or symbols, this would involve processing vehicles in virtue of their syntactic structure; while in the case of neural representations, it would involve processing vehicles in virtue of their systematic relational structure. The point is that if the input–output mapping is sensitive to at least some portion of the medium-independent vehicle over which it is defined, then it counts as a computation.

So, putting these three components together, the mechanistic account claims that concrete computation occurs wherever there is a physical system that has an organization of spatiotemporal components such that it computes an abstract function in virtue of manipulating medium-independent vehicles. As Piccinini describes the view: “Concrete computing systems are physical systems that are functionally organized to manipulate medium-independent vehicles in accordance with a rule that applies to all vehicles and depends on the medium-independent properties of the vehicles (and possibly the system’s internal states) for its application” (2015, p.5). The emphasis is on functionally integrated systems that compute at least one abstract function via vehicle manipulation.^6^

An example might help. Consider the neural network in the ocularmotor system responsible for horizontal eye movement (Robinson 1989; Leigh and Zee 2006). This system exhibits all the characteristics of a mechanistic computing system. First, the system is a causally integrated connection of spatiotemporal components (neurons) poised to produce some behaviour (eye movement). It is a functional mechanism. Second, the system computes at least one abstract function (an integration relation). It does so in virtue of preserving the relationship between eye-velocity and eye-position. Third, the system computes its function via the manipulation or transformation of medium-independent vehicles: information contained within the cortex. Morphic relations between eye-velocity and eye-position are manipulated to compute horizontal eye movement. The ocular-motor system satisfies each of the three conditions on mechanistic computation.

The mechanistic account is also distinct from causal mapping accounts on at least two fronts. First, it holds that computing systems are functional systems of a specific mechanistic type; second, it holds that computation is achieved through the use of medium-independent vehicles. Although the causal mapping account acknowledges the importance of causally integrated systems, the mechanistic account takes this condition further. This is because in addition to specifying in what ways physical structures can be processed, the mechanistic account also requires substantive organizational integration. It places more stringent conditions on when physical states can be interpreted as performing computations than the causal mapping account.

As promising as all this sounds, before the mechanistic account can serve as the basis for an updated wide computationalism, it also has to be shown that it can satisfy the six desiderata previously outlined. Consider each desideratum in turn.

First, and perhaps unsurprisingly, the mechanistic account settles the objectivity question. The specification of several conditions for concrete computation makes it a matter of fact as to whether a given physical system is a computing system – correspondingly, this also means that the account provides a solution to the problem of computational implementation.

With respect to the second desideratum, the account is successfully able to navigate the computational explanation versus modeling distinction. To see why, consider the ocular-motor example again. There, it was in virtue of the integration function that the system was able to compute horizontal eye movement. The system implemented a program or rule in the service of particular behaviour. The output of the system, horizontal eye movement, was the direct result of the function computed. One of the system’s functions is to compute eye position using an integration relation – the system instantiates a computational procedure rather than simply being described as having one.

How about desiderata (3) and (4)? Here, again, the mechanistic account is more resilient than its causal mapping counterpart. Recall that the problem for the causal mapping account was that it was unable to exclude non-paradigmatic cases, e.g., respiration, solar systems, etc. The reason was that it was too liberal in its mapping conditions; a large number of non-computing systems qualified as computational in virtue of having the right kind of causal transitions between physical states.^7^ In contrast, the mechanistic account strikes a better balance. It places further restrictions on physical computation. Consider, for example, the digestive system. Though the digestive system is appropriately described as a functional mechanism, it fails to meet an abstract functional description in terms of computing medium-independent variables. It therefore fails to qualify as a computing system. The addition of the functional mechanism and medium-independent processing requirements serves to exclude the wrong sorts of physical systems.

Fifth, the mechanistic account explains how a computing system can miscompute. This is because it focuses attention on how mechanisms can break down. Piccinini (2015, ch.7) points out that a computing mechanism is evaluable according to five perspectives: (i) how its designers intended it to compute a function, (ii) whether it is actually designed to compute a particular function but was built in such a way that it actually computes a different function, (iii) whether what was built malfunctions, (iv) whether the system is misconfigured (e.g., programed wrongly), (v) and whether the system is used incorrectly. In each case, whether through fault of designer, builder, or user, the miscomputation rests with the functional organization of the system. Functional organization is indispensable to the construction, execution and interpretation of computing systems. By requiring physical systems to be functional mechanisms, the mechanistic account successfully offers an explanation of miscomputation, because it draws attention to how the integration of spatiotemporal components drives when, how, and why physical systems miscompute.

Finally, the mechanistic account can taxonomize computing systems. This follows in virtue of its ability to distinguish between various mechanistic properties. For example, ‘being programmable’ requires having certain transducers and storage capacities. Computing systems that fail to have these properties might still compute, but may not be programmable – most Turing-machines would meet this description. By appealing to properties of functional organization, the mechanistic account takes advantage of the fact that mechanistic properties have computational implications.

It seems that the mechanistic account, at least as developed by Piccinini, provides a robust account of concrete computation. Not only does it provide a solution to the problem of implementation, but it also does so in a way that it does justice to the sciences of computation.

## Wide Mechanistic Computation

The question to consider is whether it is possible for physical computing systems of the type described by the mechanistic account to include elements outside the individual – that is, whether the mechanistic account can be squared with wide computationalism.

The answer turns out to be rather straightforward. The reason is that, similar to the casual mapping account, the mechanistic account also remains neutral about what physical parts of the world can be integrated so as to form a physical computing system. Similar to the causal mapping account, the method of computational individuation is location neutral. Whether or not a functional mechanism, one that processes medium-independent vehicles, is constituted by spatiotemporal components squarely localized within the individual or crisscrossing into the world is entirely an *a posteriori* question. Some physical computing cognitive systems might be entirely ensconced within the body, but some might as easily spread out over brain, body and world.

Piccinini even acknowledges this possibility in a footnote to his chapter on the mechanistic account: “I am officially neutral on whether the components of psychological computing mechanisms extend beyond the spatial boundaries of organisms” (2015, ch.7). Piccinini recognizes that whether psychological computing mechanisms are constituted in part by components outside the individual is an empirical question. Because the mechanistic conditions on concrete computations are medium and location neutral, the question of wide computational systems is open. The real task, then, is to show that some organisms in fact implement wide mechanistic computing systems.

There are two examples I want to draw on in establishing the empirical plausibility of wide mechanistic computation. In each case, the focus is on active sensory systems.

The first is sonar-emitting bats. Bats have become a staple example of active sensory systems since it was discovered that they hunt using self-generated acoustic signals (echolocation) (MacIver, 2009). Consider one aspect of echolocation: object detection. Bats use two different methods for object detection. On horizontal planes, bats use time and intensity differences in the returning acoustic signals to detect objects; while on vertical planes, the bats’ inner ear, the pinna-tragus, forms a pathway through which the incoming signal is filtered. The formation of the skin and supporting tissue transforms the signal into a range of spectral cues, which then get further processed neuronally.

There are two points to note about this example. First, the acoustic signal is more than just a passive input to the bats’ navigation system. The propagation of acoustic pulses actively drives obstacle detection. Second, it is the neural processing plus morphology and acoustic environment that facilitates object detection. The neuronal processing alone is insufficient. What this suggests is that the bats’ ability to detect objects along vertical planes is realized by spatiotemporal components spread out across the brain, body and world. The bats’ spatial navigation system is supported by a wide mechanism.^8^ This is the first condition on mechanistic computation.

Why view these components as forming a wide mechanism rather than casually related but distinct elements? The answer lies in the high degree of organization and structure exhibited by the components. It is only through the coordinated activity of parts spread across the brain, body and world that vertical object detection is achieved. It is not just that the acoustic signal and morphology of the bat that play a role in the delivering inputs to the internal processing. It is that both actively construct and transform the information latter used for internal processing. They are part of the underlying causal mechanism responsible for the animals’ perceptual capacity.

What about condition two? What abstract function is being computed? The answer here is one already encountered: object detection. Because there is a clear mapping from the acoustic signals outside the bat to the internal outputs (representation of objects in the vertical plane) via the simplifying structure of the *pinna-tragus*, the wide mechanism can be said to compute at least one abstract function. The difference in this case is that the input resides neither on nor in the bats’ sensory transducers. Rather, it is part of the environment. The acoustic signals on which the bats’ inner ear operates already contain information about objects in the environment.

Finally, Piccinini also claimed that a mechanism must compute a function in virtue of manipulating some portion of medium-independent vehicles. Gibson’s (1966, 1986) notion of “invariants” offers a useful starting point here. For Gibson, interactions of mechanical forces often produce systematic, structural regularities in the environment. These invariants are picked up by organism’s perceptual system. They are used to guide, sustain, and regulate behaviour. For example, because light is constantly diffused and reflected throughout the environment, there are optical textures that overlay surfaces. The amount of texture corresponds to the amount of terrain. As the density of optical texture increases, the scale of the space is revealed. Optical textures provide crucial information about the environment that an organism’s perceptual system can use to gauge distance.

Acoustic signals operate in an analogous way. Because sonic energy refracts and diffuses through gases, liquids, and solids, the arrangement of the environment and the medium of transmission shape the vibratory field in which bats navigate. It structures acoustic signals, providing important cues about object location and distance. This means that the vehicles transformed over the course of processing are external information-bearing structures. They are medium-independent vehicles that are persistent and unchanged over time and which carry information about the environment. Manipulation of the informational vehicles runs from the environment through the pinna tragus to the bat’s brain.

Notice that the external vehicles are not medium-independent in virtue of the fact that they carry information, but rather in virtue of the fact that the relevant computations are sensitive to only some portion of the informational vehicles, i.e. the invariant structure of the acoustic signal carrying information about location and direction. What matters for computation is not that the bat is responsive to the sound qua sound, but that the bat is responsive to the sound qua information-bearing structures within the sound. As Piccinini explains: “Since concrete computation and their vehicles can be defined independently of the physical media that implement them, we shall call them “medium independent” (2011, p.8). The abstract character of computational descriptions means that medium-independence follows in virtue of the relevance of specific parts of physical media to the overall computation being carried out, whether that is syntactic structure in symbols or invariant relational structure in sound.

Given the above, there seems to be good reason to think that the bats’ navigation system instantiates a wide computational system of the mechanistic variety. Not only does it involve a mechanism that spans the brain, body and world, but it also computes an abstraction function via the transformation of medium-independent vehicles. It meets all three requirements of the mechanistic account.

Animal cognition is one thing, but is there a human example? Continuing with the idea of active sensory systems, the next case to consider is spatial navigation by sightless or blind individuals.

One common assumption is that sightless individuals are at a greater disadvantage than sighted individuals during spatial navigation because of a lack of crucial visual information (Lynch 1960). Several studies have recently begun to cast doubt on this assumption, as spatial competence has been found to be increasingly less dependent on visual experience than initially thought. Ricciardi et al. (2009), for example, have shown that the sound of an action engages the mirror neuron system for action schemas, even when not learned through the visual modality.

One important take away from this research is that it suggests the use of a supramodal sensory representation in spatial navigation. This result is important because it comports well with how spatial navigation is mainly achieved within sightless individuals – that is, through the collection of spatial information via haptic and audio channels. A critical component of this collection process is the systematic feedback of haptic information through prosthetic devices, such as cranes or personal assistive devices, in addition to hands, palms and fingers – canes, for example, provide low-resolution information about the immediate environment through a semi-spherical exploratory sweeping motion. What I want to suggest is that, similar to the bats’ sonar-emitting echolocation system, the prosthetic-aided navigation system can be thought of as a wide mechanism, one that supports an abstract function through which medium-independent vehicles are manipulated. Notice how the example meets the three mechanistic conditions.

Consider the first condition. Under normal circumstances, it is undoubtedly correct to say that spatial navigation is supported by an internally constituted mechanism. However, in the case of sightless individuals, the breakdown of internal components requires recruitment of external substitutes. This is the role occupied by prosthetics such as canes or personal assistive devices, which can even include sonar-emitting devices (Lahav and Mioduser 2008). Under these conditions, the integrated spatiotemporal components form a wide mechanism. They form an integrated mechanism that spreads out beyond the neuronal.

Next, consider what function is computed by this wide mechanism. One possible answer is that the haptic information delivered by prosthetic devise aids in the construction of a “survey representation”, a disposition or layout of spatial features – direction and distance, for example (Loomis et al. 1993). The purpose of this representation is to facilitate finding trajectories or routes through the environment. Interpreted in this way, the abstract function is one that runs from the environmental signals generated from the repeated tapping of the prosthetic device to the internal spatial representation of the environment. There is a mapping of inputs I to output O via the prosthetic device.

Lastly, the spatial navigation system of sightless individuals involves manipulating medium-independent vehicles. The active exploratory strategies used by sightless individuals transform and manipulate environmental structures, particularly sonic and tactile information, in order to simplify and reduce internal processing. Much like the pinna-tragus of the bat, the prosthetic devices form morphological tools through which information is externally directed. There is a transformation of sensory information through external then internal structures. Once again, these considerations point toward the presence of a wide mechanistic computational system.

Actual examples of wide computational systems, such as the above, are important. As Segal points out in his review of Wilson: “the question of the truth of wide computationalism is a question about the proper domain of psychological theories (or at least cognitively scientific theories), it is a question about the extent of the natural phenomenon of cognition” (1997, p. 154). Without a demonstration of wide physical computing systems, the idea of wide mechanistic computation remains plausible but unsubstantiated. What I have tried to do, like Wilson and Hutchins beforehand, is show that in some cases wide computational systems are, in fact, implemented in cognizing agents.

## Objections

Time to consider some possible concerns. First, one might worry that wide computationalism, even of the mechanistic variety, fails to offer principled grounds on which to distinguish internal (brain bound) cognitive systems from wide cognitive systems. Given that a fair amount of cognitive science attempts to distinguish the underlying systems supporting psychological behaviour, wide computationalism should be able to make such an important differentiation.

Two points are worth considering here. First, recall that the computational theory of mind leaves open the possibility that some computational systems are realized by elements outside the individual. That is at least part of the moral of classic multiple realizability. Second, recall that any successful account of concrete computation is going to have to provide a taxonomy of different computing systems (desideratum six). Together, these points offer one potential answer to the ‘being principled’ concern. Since cognition is a form of computation and computation is location neutral, then insofar as one is able to identify how computational systems are implemented via the account of concrete computation offered, one has principled grounds on which to distinguish internal and wide cognitive systems. Individuating internal and wide systems simply requires identifying the right kind of conditions on physical computation.

Consider, again, the two previous examples. There, the two systems qualified as physical computing systems in virtue of meeting all three conditions of mechanistic computation. The only difference was that in the one case the system was partly constituted by parts in the world, while in the other it was completely contained within the individual. Insofar as wide computationalism relies on an account of concrete computation that is sensitive to differences in mechanistic properties, it can distinguish between computing systems instantiated both within and beyond the individual.

Consider a second concern. One debate that is near and dear to the heart of many philosophers of mind is whether cognitive states represent intrinsically (in virtue of themselves) or whether they represent in virtue of having meanings assigned to them. The question is whether the mind has ‘original’ or ‘derived’ intentionality (Searle 1980, 1983; Dretske 1981). Segal (1997) raises concerns about original intentionality in the context of wide computationalism:

Someone who claims that original intentionality is restricted to brains (or things enough like brains) and certainly not something present in pieces of paper, or even pocket calculators, is likely to be unimpressed by wide computationalism. She would likely draw a distinction between cognition proper and mere computation. Cognition proper would be restricted to systems the symbols of which are originally intentional. (p.153)

The worry is that whereas cognition trades in original content, wide computational systems trade, at least partly, in derived content. Since wide systems do not deal in the right kind of content, they should not be thought of as properly cognitive. There is a long-standing debate over original intentionality, and this is not the place to enter into the discussion. Suffice it to say, several authors have questioned the claim that original intentionality forms any sort of “mark of the cognitive” (Dennett 1987; Wilson and Clark 2009).

For present purposes, what is important is that although semantic accounts are compatible with computation, they in fact still require non-semantic individuation. Functional and structural (non-semantic) properties, such as programming language or architecture, are also going to contribute to the individuation of physical computations. Individuating computational systems by function alone – by what they represent – will often fail to individuate physical computations finely enough. As long as semantic information is built out of syntactic structure in some way, which is not only plausible but the going view of most cognitive scientists, wide computationalism is going to remain plausible. Even if it turns out that semantic accounts of computation are correct, the syntactic underpinnings of semantic information still deliver wide computationalism. Segal’s worry, then, is too quick. It fails to appreciate that computational cognition still is going to imply syntactic considerations of the kind pertinent to wide computationalism, whether or not cognition turns out to trade in original or derived content.

Finally, one might worry more generally about value of wide computationalism. Does wide computationalism have anything to add as a research strategy to cognitive science? Wilson (1994) has something like this concern in mind when he says: “the most interesting issue concerns not the coherence of wide computationalism but the extent to which a wide computational research strategy is and could be employed within cognitive psychology” (p.371).

Here is one way wide computationalism might be of wider use to cognitive science: the ongoing debate over extended cognition. Some opponents of extended cognition have suggested that environmental and bodily processes and states are too unwieldy to be brought under a framework that also contains neural processes (Adams and Aizawa 2008; Rupert 2004, 2009). The “motley crew” of extended cognition undermines its chances of developing a scientifically tractable approach to cognition. Taking wide computationalism seriously affords one answer to this challenge. This is because wide computationalism provides a framework for investigating concrete physical computing systems that cross into the world. Parts of the body and world can be integrated into and thought of in terms of performing computations, insofar as they are part of wide computational systems. Wide computationalism offers a potential rubric from within which to conduct extended cognition research. One of the potential uses of wide computationalism is its ability to link individualist forms of computational psychology with more externalist-friendly extended approaches. It offers another prospective ship on which to navigate the conceptual waters of cognitive science.

## Conclusion

Time to take stock. I set out to show that wide computationalism offered an important but overlooked option for cognitive science. I attempted to show that it offered not only a coherent and plausible account of concrete computation, but that it also found empirical support from examples in animal and human cognition. I further developed the view by defending it from several potential objections and charting its potential use for cognitive science more generally. This is by no means the final word on wide computationalism. Much more work is required -- for example, it still remains unclear how wide computationalism fits with other core concepts in cognitive science and philosophy of mind such as functionalism, intentionality or representation. Nonetheless, what the preceding discussion has done, I hope, is convey the sense that wide computationalism offers a substantive and viable supplement to existing individualistic research strategies; that it provides a plausible and theoretically fruitful avenue for cognitive science to further explore. Summatively speaking, it would not be unfair to say that the prospects of wide computationalism are looking up.

## Footnotes

1 Causal mapping accounts get cashed out in various ways. Some opt for a counterfactual approach, others a dispositional approach. The important point is that in all cases what actually ground computations is causal relations.

2 See Chemero (2011) or Varela et al. (1991) for an alternative perspective.

3 One point of difference between Wilson and Hutchins’ views lies in the breadth of the computational system identified. Wilson’s examples identify wide computational systems applying to largely brain/environment composites; while, in contrast, Hutchins broadens the reach to include individual agents. This results in a difference in emphasis when it comes to the location of representational states within each system. For Wilson, because the wide computational system runs from the formal primitives of the environment to the processing centers of the brain, the representational states are located within the brain; whereas for Hutchins, the representational states relevant to defining the navigational computation are spread out across the coordinated activity of the individual team members (see Hutchins 1995, ch.4). Though at this first glance this difference might seem notable, particularly in light of some hostility occasionally leveled by proponents of extended cognition toward internal representations, nothing crucially important hangs on it. Rather, the difference emerges largely as a result of the differing computational units emphasized within each analysis. The scope and elements of the computational system help determine the kind and location of representational states implicated.

4 This is in contrast to simple mapping accounts, such as Putnam (1967), that place no restrictions on the physical states that might form the equivalence class for computational description. This is why Putnam is skeptical of computational theories of mind.

5 This does not necessarily imply that computing system need to *represent* a rule internally, for this would overly restrict computational explanation, excluding paradigmatic cases of computing devices such as the finite state automata.

6 There is more to Piccinini’s account then what is presented here. However, this description should suffice to outline the basic features of the view. For a fuller description see Piccinini (2015, ch.7).

7 This is why science is filled with computational models. The mathematics of computation is particularly effective at describing and predicating natural phenomena (see Frigg 2012).

8 For another example of a wide mechanism see Wilson’s (2010, 2014) discussion of the giant water bug: Lethocerus.
